# Approaches to learning mathematics: preliminary evidence of a concise, valid, and reliable instrument

**DOI:** 10.3389/fpsyg.2023.1286394

**Published:** 2023-10-18

**Authors:** Yusuf F. Zakariya

**Affiliations:** ^1^Department of Science Education, Ahmadu Bello University, Zaria, Nigeria; ^2^Department of Mathematical Sciences, University of Agder, Kristiansand, Norway

**Keywords:** deep approaches to learning, higher education, R-SPQ-2F, reliability, surface approaches to learning

## Abstract

We assess students’ approaches to learning mathematics not only to predict students’ learning outcomes but also for its crucial utilities in the teaching and learning process. These utilities range from evaluating effective instructional interventions, determining students with learning difficulties, and comparing teaching and learning experience in higher education. However, measures of the constructs have raised validity concerns among researchers. A root cause of these validity concerns is traceable to the failure of these measures to account for the content-specificity of approaches to learning. Building on a previously developed general measure of the constructs, I designed this study to bridge this gap by developing and validating approaches to learning mathematics questionnaire (ALMQ). 352 first-year engineering students who gave voluntary consent participated in the study. The students were mainly males with ages ranging from 15 years to 29 years. The average age was 20.67 years, and its standard deviation was 2.164. I analysed the generated data using confirmatory factor analysis and judged the consistency of hypothesised models with the generated data using a combination of criteria. The findings revealed a two-factor ALMQ with seven items which demonstrated an excellent global and local fit of the generated data. The standardised factor loadings for all the items were above 0.68 with an average of 0.73 showing the high strengths of the items in measuring their respective constructs. I also found a reliability coefficient of 0.81 for deep approaches, 0.77 for surface approaches, and 0.72 for the two-factor ALMQ. These findings suggest preliminary evidence of the validity and reliability of ALMQ. I discussed the practical implications of the findings for educators, policymakers, and researchers interested in improving the mathematics learning experience.

## 1. Introduction

*Abdullah has amazing parents who care about his success in mathematics. However, Abdullah’s performance in mathematics turned poor shortly after joining the engineering programme of a prestigious university. His mother hired an academic counsellor to investigate what could be wrong with him. For a semester, the counsellor followed Abdullah and conducted a series of tests and interviews to gather evidence of cognitive, affective, psychomotor, and social factors that could be responsible for the poor performance. The counsellor could not find anything wrong with Abdullah in these aspects. Instead, she recommended a change of mathematics teacher for Abdullah. Without any delay, Abdullah’s mother hired a private award-winning mathematics teacher who can use various teaching methods and strategies to create an interesting learning experience for Abdullah. Yet, Abdullah’s performance in mathematics has not improved. What could be missing in this scenario?*

Mathematics for first-year undergraduate students otherwise called foundational mathematics courses is crucial not only in bridging pre-university and university mathematics experience but also in shaping higher courses in mathematics-intensive university programmes. However, an accumulation of evidence suggests that students following mathematics-intensive university programmes, e.g., science, technology, engineering, or mathematics (STEM) courses find it difficult to pass foundational mathematics courses around the world (Ellis et al., 2016; Bigotte de Almeida et al., 2021; Zakariya et al., 2022a). This poor performance in foundational mathematics courses has many consequences in higher education such as increased absenteeism, non-completion of degrees, change of majors (usually from STEM to non-STEM programmes), and development of negative attitudes towards mathematics (Ellis et al., 2016; Bigotte de Almeida et al., 2021). As a way of alleviating these consequences, researchers around the world launched investigations into the causes of poor performance in foundational mathematics and came up with areas of interest for improved performance in the courses. Among highly researched areas of interest in improving students’ performance in foundational mathematics courses concerns students’ approaches to learning in higher education (Zakariya et al., 2022b; Lahdenperä et al., 2023).

Approaches to learning are a combination of students’ dispositions to learning and strategies used while learning a given course material (Marton and Säljö, 2005; Biggs, 2012). They are deeply rooted in students’ motivation for learning a presented content which in turn determines the processes through which they go about learning the content. Thus, students who are motivated by developing a conceptual understanding of the learning content adopt approaches (deep) that are qualitatively different from approaches (surface) adopted by students who are motivated by merely passing the course. Deep approaches to learning are generally linked with high performance in mathematics while surface approaches to learning are linked with poor performance in mathematics among university students (Maciejewski and Merchant, 2016; Murphy, 2017). Apart from the influence of approaches to learning on performance in mathematics, there are interesting utilities for determining students’ approaches to learning in higher education. These include their use to evaluate effective pedagogical interventions, to determine students with difficulties in learning, and to make inter-class comparisons of teaching and learning experience (Biggs et al., 2001; Lahdenperä et al., 2019). The multiple uses of determining students’ approaches to learning not only attract researchers’ attention but also necessitate a carefully developed measure of the constructs. The present study aimed to contribute to the international body of knowledge on the measurement of approaches to learning in higher education with a particular reference to mathematics.

For more than two decades, researchers have consistently worked on the measurement of students’ approaches to learning in higher education. This work has led to the development and validation of instruments such as approaches and study skills inventory for students (ASSIST), revised approaches to studying inventory (RASI), and revised two-factor study process questionnaire (R-SPQ-2F) (Entwistle and Tait, 1994; Tait et al., 1998; Biggs et al., 2001). Researchers (e.g., Zakariya and Massimiliano, 2022) claimed that R-SPQ-2F (a 20-item instrument with equal items on each of the deep and surface subscales) is the most popular and most researched instrument for measuring students’ approaches to learning in higher education. The claim is based on the volume of publications using R-SPQ-2F and its translation and validations in many languages such as Arabic (e.g., Shaik et al., 2017), Chinese (e.g., Xie, 2014), Dutch (e.g., Stes et al., 2013), Japanese (e.g., Fryer et al., 2012), Norwegian (e.g., Zakariya, 2019), Spanish (e.g., Justicia et al., 2008), and Turkish (e.g., Önder and Besoluk, 2010). Consequently, the construct validity of R-SPQ-2F has been challenged especially when the instrument was translated into different languages. Contrary to the hypothesised factor structure of R-SPQ-2F by Biggs et al. (2001), Justicia et al. (2008) in their study concluded that neither the four-factor nor hierarchical-factor models reflect the factor structure of the instrument. Instead, they found evidence in support of a two-factor model with ten items measuring each of the deep and surface approaches to learning. Further research on R-SPQ-2F by Stes et al. (2013) recommended the removal of five items to achieve an accepted construct validity of the instrument. The construct validity problem of R-SPQ-2F has led researchers to argue for either the removal of some items or a complete revision of the instrument (Stes et al., 2013; López-Aguado and Gutiérrez-Provecho, 2018). Following recommendations of previous validity studies on R-SPQ-2F, Zakariya and Massimiliano (2022) developed and validated, through a rigorous process, a short form of the instrument that contained eight items (four items on each of deep and surface subscales of the instrument).

As far as we know, the short form of R-SPQ-2F by Zakariya and Massimiliano (2022) is the latest validity study on the measurement of students’ approaches to learning. However, both theoretical and empirical evidence suggest that approaches to learning, as well as its measurement, are sensitive to the learning context/content (Eley, 1992; Marton and Säljö, 2005), and mathematics learning, in particular (Maciejewski and Merchant, 2016; Lahdenperä et al., 2019). That is, a student can adopt deep approaches to learning a subject and simultaneously adopt surface approaches to learning another subject. This is because students’ approaches to learning depend not only on individual characteristics but also on the context that learning takes place (Biggs et al., 2001). The contextual dependence of approaches to learning makes them different from learning styles that are highly resistant to change (Murray-Harvey, 1994). Building on the contextual dependence of approaches to learning, I believe it is prudent to account for a specific subject in the measurement of the constructs. As such, I introduce approaches to learning mathematics questionnaire in this study. The purpose of this study is therefore to develop and validate approaches to learning mathematics questionnaire (ALMQ) with high psychometric properties. I believe that ALMQ will capture the context-sensitivity of the constructs which will not only increase the accuracy of the measured constructs but also unlock the potential of its utilities in evaluating effective pedagogical interventions, determining students with difficulties in learning mathematics, and making inter-class comparisons of mathematics teaching and learning experience.

## 2. Conceptual framework

### 2.1. Theoretical foundations of approaches to learning

For more than four decades, several theoretical perspectives have been put forth to conceptualise and operationalise approaches to learning in higher education. These theoretical perspectives include information processing theory (Schmeck et al., 1977; Moreno and DiVesta, 1991), phenomenology (Marton and Säljö, 1976; Prosser and Millar, 1989), and system theory (Entwistle and Waterston, 1988; Biggs, 1993). A crucial distinction between these theoretical perspectives is whether approaches to learning are mainly individual characteristics (as in information processing theory) or they are dependent on the teaching-learning context. Be that as it may, these theoretical perspectives have contributed to the formulation of students’ approaches to learning (SAL) theory which views approaches to learning as an emergent of a combination of both individual characteristics and the context in which learning takes place (Biggs et al., 2001; Entwistle, 2005; Marton and Säljö, 2005). In the SAL framework, therefore, approaches to learning are strategies or adopted processes that emerge from differing intentions of students for learning presented content. Within the context of mathematics learning, I can conceptualise approaches to learning as adopted processes or strategies used by students while learning mathematics that are induced by students’ intentions for engaging with mathematical content.

Research shows that students’ intentions for engaging with learning content may be to understand the learning content as much as possible, to cope with the course requirements, and to optimise course grades (Biggs et al., 2001; Entwistle, 2005). These intentions lead to the characterisation of approaches to learning in SAL theory into the deep, surface, achieving/strategic approaches to learning (Biggs et al., 2001; Entwistle, 2005). That is, students with deep approaches to learning are motivated by their intention to develop a proper understanding of the learning content. They do this by relating new ideas with prior knowledge, searching for underlying patterns and meanings, and engaging actively with the learning content (Entwistle, 2005). Students with surface approaches to learning, on the other hand, are motivated by their intention to cope with content requirements with minimal work done. They do this by using rote learning as a tool to reproduce the learning content, engaging with the learning content as unrelated pieces of knowledge, and being disinterested in either reflecting or searching for underlying meanings of the learning content. It is crucial to remark that the use of memorisation techniques is not peculiar to students using surface approaches to learning. Rather, students with deep approaches to learning can also use memorisation techniques. The difference is that the former use memorisation techniques selectively and to reproduce the learning content while the latter use the techniques as a tool to develop conceptual understanding. For instance, students with deep approaches to learning can memorisation a procedure that will be used in proving a theorem in mathematics.

As for students with strategic approaches to learning, they are motivated by their intention to obtain the highest possible grade in the course. They do this by managing their time effectively, working consistently throughout the semester, and studying required materials for assessment (Entwistle, 2005). The deep and surface approaches to learning characterise students’ actual engagement with the content either by meaning-making or by reproducing the content, respectively. Meanwhile, the strategic approaches to learning characterise students’ organisation of time and space of content engagement. Therefore, researchers (e.g., Biggs et al., 2001; Zakariya et al., 2021) have argued that deep and surface approaches to learning sufficiently characterise the constructs while strategic approaches to learning can simultaneously be both deep and surface. That is, students with deep approaches to learning can be strategic by organising time and space while students with surface approaches to learning can also do the same. These arguments were founded on previous empirical studies (e.g., Wong et al., 1996; Kember et al., 1999) that showed that items hypothesised to measure strategic approaches to learning sufficiently load on either deep or surface approaches to learning. Following the line of thought of these researchers and to avoid ambiguity in the measurement of the constructs, I characterise approaches to learning mathematics as either deep or surface approaches. This is done such that students can either adopt deep approaches to learning mathematical content or surface approaches to learning the content at a given point in time.

Another crucial theoretical foundation of approaches to learning is that the constructs are context-dependent (Biggs et al., 2001; Marton and Säljö, 2005). There is an accumulation of both theoretical and empirical evidence that suggests a few contextual factors that influence students’ approaches to learning. These contextual factors include the nature of the learning materials, forms of assessment, teaching-learning environment, and instructional methods (Biggs et al., 2001; Marton and Säljö, 2005; Maciejewski and Merchant, 2016; Lahdenperä et al., 2019). By implications, students’ approaches to learning change in response to changes in these contextual factors. For instance, an undergraduate student may adopt deep approaches to learning for a core course in mathematics while using surface approaches to learn an elective course within the same programme. The fact that one course is core and the other is elective could stimulate different intentions for learning the courses leading to different approaches to learning by the same student. Maciejewski and Merchant (2016) provide an empirical basis to support this argument by showing that students’ approaches to learning change with changes in mathematical tasks across different levels of university education. In a similar manner, students following a course with a formative form of assessment are likely to adopt a different approach to learning from students following a course with a summative form of assessment. Biggs (2012) showed that the formative form of assessment encouraged deep approaches to learning while the summative form of assessment encouraged surface approaches to learning.

More so, evidence suggests that approaches to learning are dependent on the teaching-learning environment which includes classroom climate, teacher-student relationship, and curriculum demands (Eley, 1992; Biggs et al., 2001; Baeten et al., 2010). More recently, Lahdenperä et al. (2019) showed that an instructional method that is student-centred supports deep approaches to learning and discourages surface approaches to learning among university students in mathematics classrooms. The dependence of approaches to learning on contextual factors creates an opportunity of using approaches to learning to examine these contextual factors. For instance, the prevalence of deep approaches to learning in a classroom could suggest that the instructional method is student-centred, the assessment method is formative, and the classroom climate promotes healthy teacher-student relationships. Apart from the contextual factors, SAL theory acknowledges the influence of motivational and affective constructs on students’ approaches to learning. These factors include students’ values, motivation, aspirations, interests, self-efficacy, and attitudes towards the course, in particular, mathematics (Marton and Säljö, 2005; Baeten et al., 2010; Zakariya et al., 2020, 2022b). The findings of these studies provide empirical support to conceptualising approaches to learning as emergent constructs from a dynamic relationship between individual and contextual factors as postulated in SAL theory.

### 2.2. Measuring approaches to learning

The development and validation of measures of approaches to learning have attracted huge attention from researchers around the world with much more concentration in higher education. Consequently, several measures (e.g., ASSIST, RASI, and R-SPQ-2F) have been developed and validated, in some instances, cross-cultural validated in different contexts (Entwistle and Tait, 1994; Tait et al., 1998; Biggs et al., 2001; Zakariya, 2019). Among these measures, R-SPQ-2F stands out in terms of its alignment with SAL theoretical postulations, its high psychometric properties, its relatively small number of items, and the high volume of research related to the measure. However, R-SPQ-2F has equally been criticised for its lack of construct validity when used in different cultural contexts (Zakariya, 2019). Historically, R-SPQ-2F is a product of a series of studies that involved conceptualisation, re-conceptualisation, operationalisation, re-operationalisation, and validation of a 42-item study process questionnaire by Biggs (1987). The 42-item study process questionnaire was re-evaluated and reduced to 20-item R-SPQ-F with 10 items measuring deep approaches to learning and 10 items measuring surface approaches to learning (Biggs et al., 2001). The 10 items are further theorised to measure motives and strategies for the two approaches. That is, R-SPQ-2F has 20 items with five items each measuring deep motive, deep strategy, surface motive, and surface strategy, respectively (Biggs et al., 2001).

There have been several calls from international researchers for a revision of R-SPQ-2F with suggestions for deleting some items of R-SPQ-2F to achieve sufficient construct validity. Following these calls, Zakariya and Massimiliano (2022) introduced a short form of R-SPQ-2F (SF-R-SPQ-2F) and investigated its construct validity and reliability in two European countries (Norwegian and Italian). They did this through a series of studies involving both Norwegian and Italian undergraduate students and confirmed that SF-R-SPQ-2F is a valid and reliable measure of approaches to learning in the two cultural contexts (Zakariya and Massimiliano, 2022). The SF-R-SPQ-2F contains eight items with four items each measuring deep and surface approaches to learning, respectively. The four items for each dimension coincide with theorised motives and strategies for the two approaches as in the original R-SPQ-2F. That is, SF-R-SPQ-2F has eight items with two items each measuring deep motive, deep strategy, surface motive, and surface strategy, respectively (Zakariya and Massimiliano, 2022). Meanwhile, SF-R-SPQ-2F items, just the like original R-SPQ-2F, are generically worded without acknowledging the context-dependence of the constructs. In this study, I extend the work by Zakariya and Massimiliano (2022) by rewording items of SF-SPQ-2F to introduce approaches to learning mathematics questionnaire and validate it in an English-speaking country. I am motivated to introduce this questionnaire by both theoretical and empirical evidence that suggests the context-dependence of approaches to learning in higher education.

## 3. Methods

### 3.1. Participants

The participants of this study comprised first-year undergraduate students following engineering programmes in the largest university in sub-Saharan Africa located in Northern Nigeria. The sampling procedure for this study was purposive as I focused on students enrolled in foundational mathematics courses. Automatically, first-year engineering students became the focus of this study because they formed the largest population of students enrolled in foundational mathematics courses at the university. A total of 352 students voluntarily gave consent to participate in the study. The students are following agricultural, chemical, civil, computer, electrical, mechanical, mining and mineral, polymer and textile, water resources and environmental engineering programmes. Considering the small number of items of SF-R-SPQ2F and the associated parameters, a sample 352 is sufficient for confirmatory factor analysis (Brown, 2015). The youngest among the students was 15 years old while the oldest was 29 years old and the average age of the sample was 20.67 years with a standard deviation of 2.164.

### 3.2. Materials

I adapted SF-R-SPQ-2F by Zakariya and Massimiliano (2022) to the mathematics context. I did this by rewording item statements of the measure to reflect mathematics. I kept the theoretical structure of the measure intact with two items each measuring deep motive, dee strategy, surface motive, and surface strategy. This process gave the ALMQ that I used in this to generate the research data. The questionnaire requires the participants to report their level of agreement to its item statements by ticking a box out of the five options from “*never or only rarely true of me*” through “*true of me about half the time*” to “*always or almost always true of me*.” Table 1 presents item wordings of both SB-R-SPQ-2F and the corresponding ALMQ.

**Table 1:** Source of item wordings for approaches to learning mathematics questionnaire.

	SB-R-SPQ-2F	ALMQ
Deep motive	I feel that virtually any topic can be highly interesting once I get into it	ALMQ01: I feel that virtually any topic in mathematics can be highly interesting once I get into it
	I work hard at my studies because I find the material interesting	ALMQ04: I work hard to study mathematics because I find the material interesting
Deep strategy	I find most new topics interesting and often spend extra time trying to obtain more information about them	ALMQ02: I find most new topics in mathematics interesting and often spend extra time trying to obtain more information about them
	I test myself on important topics until I understand them completely	ALMQ03: I test myself on important topics in mathematics until I understand them completely
Surface motive	I find it is not helpful to study topics in depth. It confuses and wastes time when all you need is a passing acquaintance with topics	ALMQ06: I find it is not helpful to study mathematics topics in depth. It confuses and wastes time when all you need is a passing acquaintance with topics in mathematics
	I see no point in learning material which is not likely to be in the examination	ALMQ08: I see no point in learning mathematics material which is not likely to be in the examination
Surface-strategy	I generally restrict my study to what is specifically set as I think it is unnecessary to do anything extra	ALMQ05: I generally restrict my study to what is specifically set as I think it is unnecessary to do anything extra in mathematics
	I believe that lecturers should not expect students to spend significant amounts of time studying material everyone knows will not be examined	ALMQ07: I believe that teachers should not expect students to spend significant amounts of time studying mathematics material everyone knows will not be examined

### 3.3. Data collection and analysis

Following a cross-sectional survey research design, we administered the questionnaire during our class visits to mathematics lecture theatres. We sought permission from course lecturers before visiting their classes and we administered the questionnaires at the end of lectures. We visited three classes dedicated to teaching foundational mathematics courses for engineering students. We properly informed the students about the aims and objectives of the research and encouraged them to participate as they formed crucial stakeholders for the success of the project. We let them know that participation in the study is voluntary, anonymous, and it attracts neither reward nor penalty in any form for participants. Following the introductory statements, we distributed the questionnaires which were completed using pen and paper by those who consent to take part in the study. The questionnaire administration including introductory statements took 12 min. After the questionnaire administration, we coded participants’ responses in Microsoft Excel and prepare the data for analysis.

I analysed the generated data using descriptive statistics involving means, variance, and correlations. I explored the data for normal distribution and found that the data contained neither excess kurtosis nor skewness because the absolute values of both statistics are less than 2. Missing values were at random and were not than 1% on all variables which pose no challenge for the analysis. Then, I used confirmatory factor analysis as inferential statistics to investigate the consistency of the measurement model of ALMQ with our generated data. Despite the normally distributed nature of the data as revealed in the exploration analysis, I treated the data as categorical and used the weighted least square mean and variance adjusted (WLSMV) estimator to account for the ordinal nature of the data. I considered a model to exhibit an excellent global fit of the data using a combination of strict criteria: the chi-square value is not significant, comparative fit index (CFI) and Tucker-Lewis index (TLI) are greater than 0.95 (Hu and Bentler, 1999), standardised root mean square residual (SRMR) is less than 0.60, and root mean square error of approximation (RMSEA) is less than 0.08 (Brown, 2015). Further, I complement the global fit of the data with the local fit by ensuring that standardised factor loadings are greater than 0.60 for each item. I performed all the statistical analyses in the Mplus programme version 8.4.

## 4. Results

### 4.1. Data exploration

The first set of results concerns an exploration of the generated data. Table 2 shows the sample size after excluding the missing values, mean, variance, skewness, and kurtosis for each item of ALMQ.

**Table 2:** Descriptive statistics of ALMQ items.

Item	Sample size	Mean	Variance	Skewness	Kurtosis
ALMQ01	352	3.349	2.074	−0.271	−1.378
ALMQ02	351	3.145	1.765	−0.063	−1.198
ALMQ03	352	3.205	1.862	−0.104	−1.264
ALMQ04	351	3.117	1.773	−0.113	−1.154
ALMQ05	350	2.614	1.757	0.452	−0.950
ALMQ06	352	2.114	1.800	0.964	−0.347
ALMQ07	351	2.239	1.869	0.761	−0.728
ALMQ08	352	2.168	1.912	0.897	−0.569

The presented descriptive statistics in Table 2 confirmed that the generated data contained neither excess skewness nor kurtosis which indicates that the data are normally distributed. Meanwhile, I generated a polychoric correlation matrix of the data instead of a Pearson correlation matrix to account for the categorical nature of the data. Table 3 presents the polychoric correlation matrix which may be used to reproduce the subsequent findings in an independent study.

**Table 3:** Polychoric correlation matrix of items of ALMQ.

	ALMQ01	ALMQ02	ALMQ03	ALMQ04	ALMQ05	ALMQ06	ALMQ07	ALMQ08
ALMQ01	1.000							
ALMQ02	0.542	1.000						
ALMQ03	0.479	0.509	1.000					
ALMQ04	0.489	0.574	0.533	1.000				
ALMQ05	−0.042	−0.039	−0.009	0.076	1.000			
ALMQ06	−0.180	−0.149	−0.220	−0.181	0.348	1.000		
ALMQ07	−0.150	−0.173	−0.197	−0.147	0.356	0.542	1.000	
ALMQ08	−0.198	−0.258	−0.223	−0.201	0.261	0.491	0.558	1.000

The presented results in Table 3 show that correlations between some items are positive while others are negative. These are expected because of the two opposing dimensions of ALMQ. I expect correlations within items measuring the same dimension (Deep approaches: ALMQ01-ALMQ04; surface approaches: ALMQ05-ALMQ08) to be positive and between dimensions to be negative. Contrary to my expectations, the correlation between ALMQ04 and ALMQ05 is positive. This is evidence of multicollinearity, and it suggests that one of these items is not measuring only what it is purported to measure. I explored this observation further in the next section.

### 4.2. Evaluation of the measurement model and the reliability

**Figure 1:**
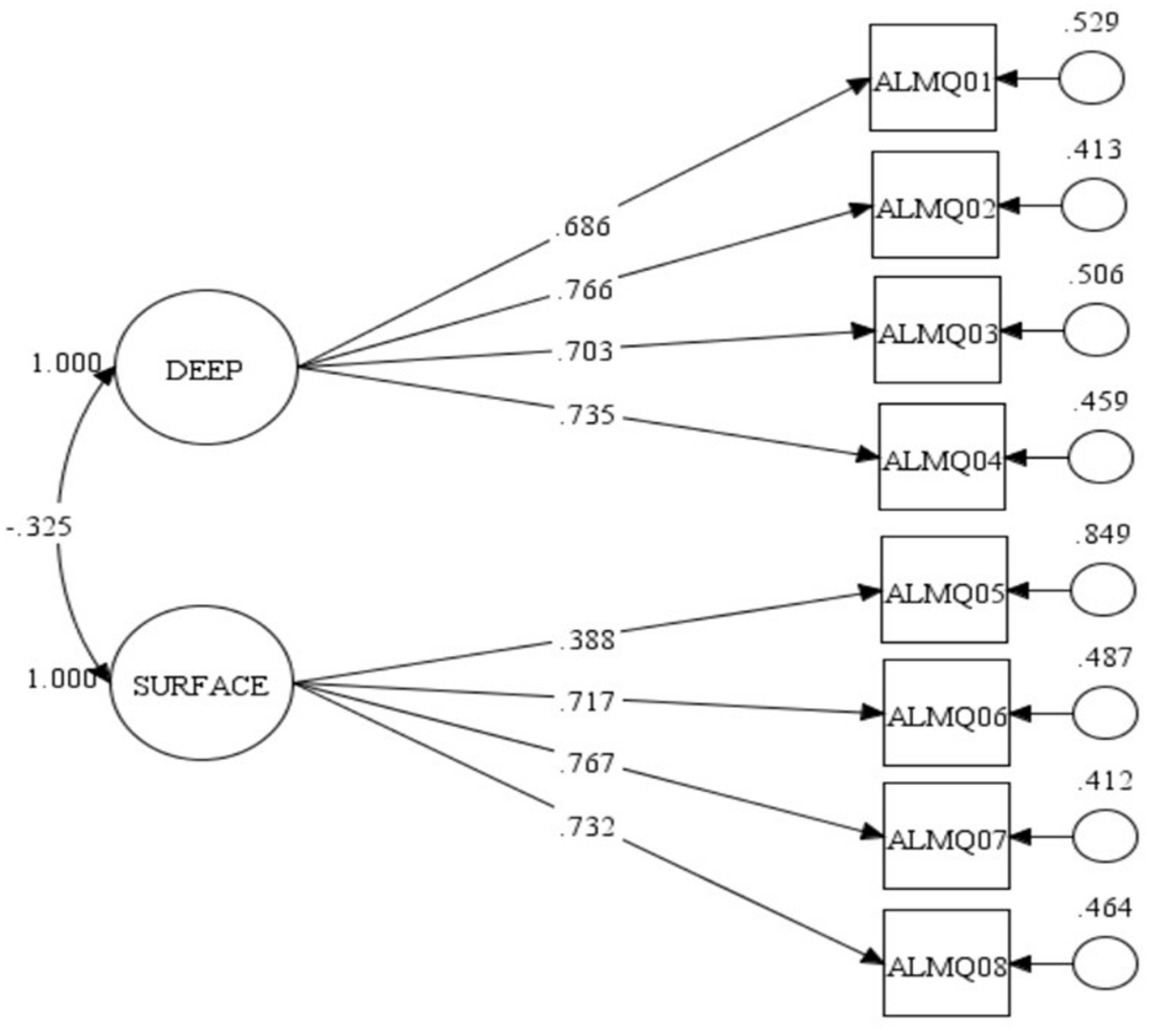
Evaluated model of eight-item ALMQ.

Following the recommendation of a two-factor measurement model by Zakariya and Massimiliano (2022), I evaluated the consistency of the model with the generated data for ALMQ. The global fit statistics show an appropriate fit of the model with generated data. The chi-square value = 33.082, *p* = 0.024, CFI = 0.986, TLI = 0.979, SRMR = 0.032, and RMSEA = 0.046 with a 90 per cent confidence interval (C.I.) of [0.017–0.071, *p* = 0.570]. These statistics demonstrate an appropriate fit of the model despite the significant chi-square. This is because chi-square values are sensitive to large sample sizes (Brown, 2015). I then proceed to examine the local fit of the evaluated model with the generated data. Figure 1 presents standardised local fit statistics of the evaluated model.

**Figure 2:**
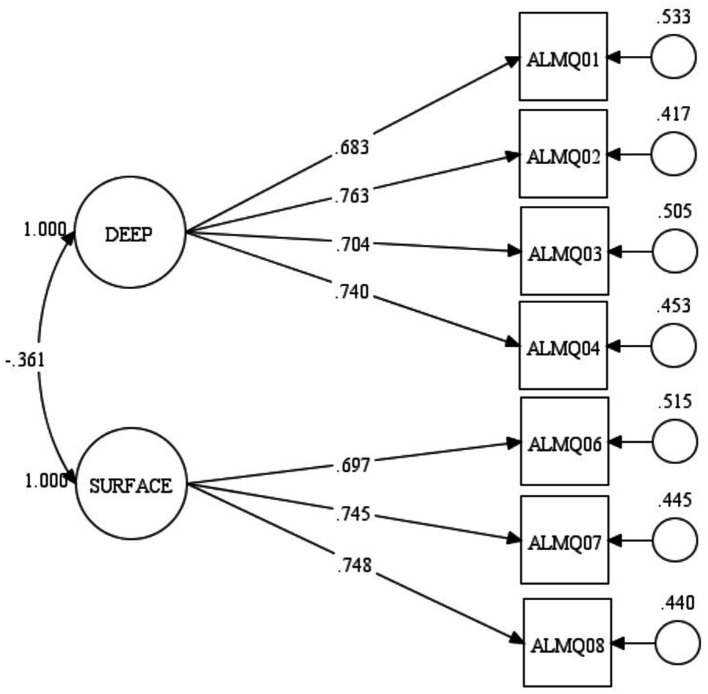
Evaluated model of seven-item ALMQ.

Figure 1 shows the factor structure of the model and the corresponding standardised factor loading of each item of ALMQ. The factor correlation (r = −325, *p* = 0.001) between deep and surface approaches to learning mathematics is negative as expected. This is true because students who scored high on the deep approaches to learning dimension of ALMQ are expected to score low on its surface approaches to learning dimension. All the factor loadings are greater than 0.60 except for ALMQ05 with a factor loading of 0.388. This means that ALMQ05 (*I generally restrict my study to what is specifically set as I think it is unnecessary to do anything extra in mathematics*) measured surface approaches to learning mathematics with weak strength. This finding is consistent with the evidence of multicollinearity as revealed in Table 3 and called for a revision of the ALMQ model. Further, Figure 1 shows the corresponding residual of each item with an exceptionally high value for ALMQ05. After considering statistical (as revealed in modification indices) and conceptual evidence to rectify the model, we decided to remove ALMQ05 from the model. The global fit statistics of the modified model of ALMQ showed an excellent global model fit with the generated data. The chi-square value = 9.664, *p* = 0.721, CFI = 1.000, TLI = 1.000, SRMR = 0.017, and RMSEA = 0.001 with a 90 per cent confidence interval (C.I.) of [0.000–0.041, *p* = 0.938]. Figure 2 presents standardised local fit statistics of the evaluated modified model.

The presented results in Figure 2 show a clear factor structure of the modified ALMQ with seven items. Appendix A shows the polychoric correlation matrix of the modified model for reproducing the subsequent findings in an independent study while Appendix B shows the final version of ALMQ. The factor correlation (*r* = −361, *p* = 0.001) between deep and surface approaches to learning is negative as expected. All the standardised factor loadings are greater than 0.60 with an average value of 0.726. These values confirm the strength of the relationship between the items and hypothesised constructs they are purported to measure. Consequently, the residuals are within acceptable values. Further, the four items (ALMQ01-ALMQ04) explain 87.70% variance of deep approaches to learning mathematics while the three items (ALMQ06-ALMQ07) explain 94.20% variance of surface approaches to learning mathematics according to the unstandardised results. The excellent local and global fits of the modified model with generated data suggest evidence of the construct validity of ALMQ. That is, ALMQ measures the constructs it is hypothesised to measure. Further, I used the presented results in Figure 2 to compute coefficient omega (Raykov and Marcoulides, 2016) as an estimate of the reliability of ALMQ. I did not use Cronbach alpha as an estimate of the reliability because of possible violations of its strict assumptions (Zakariya, 2022). The choice of omega is as well consistent with previous studies (McNeish, 2018; Hayes and Coutts, 2020) that have advocated cautions with using Cronbach’s alpha to estimate the reliability coefficient of research instruments. I found that the reliability coefficient for the deep dimension was 0.814, the surface dimension was 0.774, and the two-factor ALMQ was 0.723. These values suggest that ALMQ is not only a valid measure of approaches to learning mathematics, but it is also reliable.

## 5. Discussion of findings

Approaches to learning are crucial constructs in the teaching and learning process especially in higher education. The relevance of approaches to learning lies in their ability to influence learning outcomes, judge the effectiveness of instructional interventions, identify students with learning difficulties, and make comparisons between teaching contexts (Biggs et al., 2001). In this study, I exposed a knowledge gap in the literature that pertains to a lack of measures of approaches to learning with a particular reference to mathematics contexts. To bridge this gap, I took insights from a carefully developed general measure of approaches to learning to develop and validate a measure of approaches to learning mathematics. Through a combination of theoretical and statistical arguments, I introduced a seven-item approaches to learning mathematics questionnaire with four items measuring deep approaches to learning mathematics and three items measuring surface approaches to learning mathematics. The findings are consistent with the findings of previous studies in many ways.

The two dimensions of the measure coincide with theoretical postulations of SAL theory as advocated by Biggs et al. (2001); Marton and Säljö (2005) and they discriminate consistently with a standardised negative correlation of −0.361 as in previous studies (e.g., Xie, 2014; López-Aguado and Gutiérrez-Provecho, 2018; Zakariya and Massimiliano, 2022). This finding constitutes evidence of the discriminant validity of ALMQ. In addition, the finding is conceptually plausible because engineering students who scored high on deep approaches to learning mathematics items are expected to score low on surface approaches to learning mathematics items. Unlike SB-R-SPQ-2F by Zakariya and Massimiliano (2022) which contains eight items, ALMQ contains seven items. With the growing interest in developing brief questionnaires in the international context, the seven-item ALQM has advantages such as less time to complete it which will reduce respondents’ burden, ease of scoring for efficient interpretation, and high psychometric properties. The evidence of construct validity of ALMQ found in the present study is consistent with previous studies on general measures of students’ approaches to learning (e.g., Xie, 2014; López-Aguado and Gutiérrez-Provecho, 2018; Zakariya and Massimiliano, 2022).

Similarly, I also found evidence of reliability for each item of ALMQ, its dimensions, as well as the whole measure. The high factor loadings (standardised average = 0.726) of the items of ALMQ with corresponding low residuals (standardised average = 0.473) provide evidence of the reliability of each of the items. In addition to evidence of the reliability, the high factor loadings indicate high strengths with which each item measures the construct it is purported to measure. The deep approaches to learning dimension of ALMQ has a reliability coefficient of 0.814 while its surface approaches to learning dimension has a reliability coefficient of 0.774. The reliability coefficients are higher than previously reported coefficients of reliability in related literature (e.g., Xie, 2014; López-Aguado and Gutiérrez-Provecho, 2018; Zakariya and Massimiliano, 2022). Comparable with previous studies (Zakariya, 2019), I also found a reliability coefficient of 0.723 for the two-dimension ALMQ. Following these findings, I conclude that ALMQ does not only measure the constructs it is purported to measure but also measures the constructs consistently. Thus, I can identify three major contributions of the present study to the literature as follows. Firstly, this study introduces a brief measure of students’ approaches to learning that considers the context of mathematics learning which has been lacking in the literature. Secondly, this study provides tentative evidence of the validity and reliability of a newly introduced measure of approaches to learning mathematics which will bolster the confidence of mathematics education stakeholders to use ALMQ in the context of mathematics learning. More importantly, the valid and reliable measure of approaches to learning mathematics reported in this study provides a crucial missing link in Abdullah’s vignette. This is because Abdullah’s mother focused on who her son is by hiring an academic counsellor to investigate his factors. The academic counsellor, on the other hand, focused on what Abdullah’s teacher does in the classroom by recommending a change of mathematics teacher. Both missed what Abdullah does in mathematics classrooms, in terms of how he goes about his learning and the strategies used while learning. With the development of ALMQ we can now appropriately quantify, with high precision, the approaches used by Abdullah while learning mathematics. This attempt could solve Abdullah’s problem and other students like following mathematics.

For the use of ALMQ in an authentic context, I recommend that researchers and classroom teachers should compute averages of the corresponding items of deep and surface approaches to learning mathematics to make composite scores for the two dimensions of ALMQ. Alternatively, if the sample size is large enough (at least 200 cases), researchers can examine the CFA of ALMQ and generate factor scores for its two dimensions. Both composite and factor scores of ALMQ can be used to predict variables such as learning outcomes in mathematics, cognitive factors, and affective factors. These scores can also be used to examine individual differences in cognitive, affective, and learning experience among students. For classification purposes, researchers can subtract the composite/factor score of surface approaches to learning dimension from the deep approaches to learning dimension for each participant. If the difference is positive the student may be classified as a deep approach learner, negative, the student may be classified as a surface approach learner, and undecided if the difference is zero. Further, researchers can use the item-level model of ALMQ to evaluate the relationship between approaches to learning mathematics and other constructs in the structural equation modelling environment. Despite the theoretical alignment and psychometric properties of ALMQ, I admit there are some limitations to this study. However, these limitations open opportunities for further research in the area of students’ approaches to learning mathematics. For instance, the evidence of the validity of ALMQ does not include external validation in the form of predictive validity by relating approaches to learning mathematics with other constructs. Also, I did not investigate the measurement invariance of ALMQ which could permit mean comparisons of the constructs across different populations and contexts. Further, this study is limited to first-year engineering students learning mathematics in higher education. This may impede the generalisation of the findings beyond this context. Put together, these limitations necessitate further research areas in predictive validity and measurement invariance using a variety of student populations in different cultures. Among other things, I call on researchers across the globe to conduct further research on ALMQ in these research areas.

## Data availability statement

The raw data supporting the conclusions of this article will be made available by the authors, without undue reservation.

## Ethics statement

The studies involving humans were approved by Ahmadu Bello University Ethics Committee. The studies were conducted in accordance with the local legislation and institutional requirements. The participants provided their written informed consent to participate in this study.

## Author contributions

YZ: Conceptualization, Data curation, Investigation, Methodology, Validation, Writing – original draft, Writing – review & editing.

## References

[ref1] Baeten M. Kyndt E. Struyven K. Dochy F. (2010). Using student-centred learning environments to stimulate deep approaches to learning: factors encouraging or discouraging their effectiveness. Educ. Res. Rev. 5, 243–260. doi: 10.1016/j.edurev.2010.06.001

[ref2] Biggs J. B. (1987). Student approaches to learning and studying. Australian Council for Educational Research. Camberwell, VIC.

[ref3] Biggs J. B. (1993). What do inventories of students’ learning processes really measure? A theoretical review and clarification. Br. Educ. Res. J. 63, 3–19. doi: 10.1111/j.2044-8279.1993.tb01038.x 8466833

[ref4] Biggs J. B. (2012). What the student does: teaching for enhanced learning. High. Educ. Res. Dev. 31, 39–55. doi: 10.1080/07294360.2012.642839

[ref5] Biggs J. B. Kember D. Leung D. Y. P. (2001). The revised two factor study process questionnaire: R-SPQ-2F. Br. J. Educ. Psychol. 71, 133–149. doi: 10.1348/000709901158433, PMID: 11307705

[ref6] Bigotte de Almeida M. E. Queiruga-Dios A. Cáceres M. J. (2021). Differential and integral calculus in first-year engineering students: a diagnosis to understand the failure. Mathematics 9:61. doi: 10.3390/math9010061

[ref7] Brown T. A. (2015). Confirmatory factor analysis for applied research. The Guilford Press, New York City.

[ref8] Eley M. G. (1992). Differential adoption of study approaches within individual students. High. Educ. 23, 231–254. doi: 10.1007/BF00145015

[ref9] Ellis J. Fosdick B. K. Rasmussen C. (2016). Women 1.5 times more likely to leave STEM pipeline after calculus compared to men: lack of mathematical confidence a potential culprit. PLoS One 11:e0157447. doi: 10.1371/journal.pone.0157447, PMID: 27410262 PMC4943602

[ref10] Entwistle N. (2005). “Contrasting perspectives on learning” in The experience of learning: Implications for teaching and studying in higher education. eds. Marton F. Hounsell D. Entwistle N.. 3rd ed (Edinburgh: University of Edinburgh, Centre for Teaching, Learning and Assessment), 106–125.

[ref11] Entwistle N. Tait H. (1994). The revised approaches to studying inventory. Centre for Research into Learning and Instruction, University of Edinburgh. Edinburgh.

[ref12] Entwistle N. Waterston S. (1988). Approaches to studying and levels of processing in university students. Br. J. Educ. Psychol. 58, 258–265. doi: 10.1111/j.2044-8279.1988.tb00901.x

[ref13] Fryer L. K. Ginns P. Walker R. A. Nakao K. (2012). The adaptation and validation of the CEQ and the R-SPQ-2F to the Japanese tertiary environment. Br. J. Educ. Psychol. 82, 549–563. doi: 10.1111/j.2044-8279.2011.02045.x 23025392

[ref14] Hayes A. F. Coutts J. J. (2020). Use omega rather than Cronbach’s alpha for estimating reliability. But …. Commun. Methods Meas. 14, 1–24. doi: 10.1080/19312458.2020.1718629

[ref15] Hu L. t. Bentler P. M. (1999). Cutoff criteria for fit indexes in covariance structure analysis: conventional criteria versus new alternatives. Struct. Equ. Model. Multidiscip. J. 6, 1–55. doi: 10.1080/10705519909540118

[ref16] Justicia F. Pichardo M. C. Cano F. Berbén A. B. G. De la Fuente J. (2008). The revised two-factor study process questionnaire (R-SPQ-2F): exploratory and confirmatory factor analyses at item level. Eur. J. Psychol. Educ. 23, 355–372. doi: 10.1007/BF03173004

[ref17] Kember D. Wong A. Leung D. Y. P. (1999). Reconsidering the dimensions of approaches to learning. Br. J. Educ. Psychol. 69, 323–343. doi: 10.1348/000709999157752

[ref18] Lahdenperä J. Postareff L. Rämö J. (2019). Supporting quality of learning in university mathematics: a comparison of two instructional designs. Int. J. Res. Undergrad. Math. Educ. 5, 75–96. doi: 10.1007/s40753-018-0080-y

[ref19] Lahdenperä J. Rämö J. Postareff L. (2023). Contrasting undergraduate mathematics students’ approaches to learning and their interactions within two student-centred learning environments. Int. J. Math. Educ. Sci. Technol. 54, 687–705. doi: 10.1080/0020739x.2021.1962998

[ref20] López-Aguado M. Gutiérrez-Provecho L. (2018). Checking the underlying structure of R-SPQ-2F using covariance structure analysis. Cult. Educ. 30, 105–141. doi: 10.1080/11356405.2017.1416787

[ref21] Maciejewski W. Merchant S. (2016). Mathematical tasks, study approaches, and course grades in undergraduate mathematics: a year-by-year analysis. Int. J. Math. Educ. Sci. Technol. 47, 373–387. doi: 10.1080/0020739x.2015.1072881

[ref22] Marton F. Säljö R. (1976). On qualitative differences in learning I: outcome and process. Br. J. Educ. Psychol. 46, 4–11. doi: 10.1111/j.2044-8279.1976.tb02980.x

[ref23] Marton F. Säljö R. (2005). “Approaches to learning” in The experience of learning: Implications for teaching and studying in higher education. eds. Marton F. Hounsell D. Entwistle N.. 3rd ed (Edinburgh: University of Edinburgh, Centre for Teaching, Learning and Assessment), 39–58.

[ref24] McNeish D. (2018). Thanks coefficient alpha, we'll take it from here. Psychol. Methods 23, 412–433. doi: 10.1037/met0000144 28557467

[ref25] Moreno V. DiVesta F. J. (1991). Cross-cultural comparisons of study habits. J. Educ. Psychol. 83, 231–239. doi: 10.1037/0022-0663.83.2.231

[ref26] Murphy P. E. L. (2017). “Student approaches to learning, conceptions of mathematics, and successful outcomes in learning mathematics” in Success in higher education. eds. Wood L. N. Bryer Y. A. (Singapore: Springer), 75–93.

[ref27] Murray-Harvey R. (1994). Learning styles and approaches to learning: distinguishing between concepts and instruments. Br. J. Educ. Psychol. 64, 373–388. doi: 10.1111/j.2044-8279.1994.tb01110.x

[ref28] Önder I. Besoluk S. (2010). Adaptation of revised two factor study process questionnaire (R-SPQ-2F) to Turkish. Educ. Sci. 35, 55–67.

[ref29] Prosser M. Millar R. (1989). The «how» and «what» of learning physics. Eur. J. Psychol. Educ. 4, 513–528. doi: 10.1007/BF03172714

[ref30] Raykov T. Marcoulides G. A. (2016). Scale reliability evaluation under multiple assumption violations. Struct. Equ. Model. Multidiscip. J. 23, 302–313. doi: 10.1080/10705511.2014.938597

[ref31] Schmeck R. R. Ribich F. Ramanaiah N. (1977). Development of a self-report inventory for assessing individual differences in learning processes. Appl. Psychol. Meas. 1, 413–431. doi: 10.1177/014662167700100310

[ref32] Shaik S. A. Almarzuqi A. Almogheer R. Alharbi O. Jalal A. Alorainy M. (2017). Assessing Saudi medical students learning approach using the revised two-factor study process questionnaire. Int. J. Med. Educ. 8, 292–296. doi: 10.5116/ijme.5974.7a06, PMID: 28829331 PMC5572421

[ref33] Stes A. De Maeyer S. Van Petegem P. (2013). Examining the cross-cultural sensitivity of the revised two-factor study process questionnaire (R-SPQ-2F) and validation of a Dutch version. PLoS One 8:e54099. doi: 10.1371/journal.pone.0054099, PMID: 23342085 PMC3546932

[ref34] Tait H. Entwistle N. J. McCune V. (1998). “ASSIST: a re-conceptualisation of the approaches to studying inventory” in Improving students as learners (pp. 262–271). ed. Rust C. (Oxford: Oxford Brookes University)

[ref35] Wong N. Y. Lin W. Y. Watkins D. (1996). Cross-cultural validation of models of approaches to learning: an application of confirmatory factor analysis. Educ. Psychol. 16, 317–327. doi: 10.1080/0144341960160308

[ref36] Xie Q. (2014). Validating the revised two-factor study process questionnaire among Chinese university students. Int. J. Educ. Psychol. Assess. 16, 4–20.

[ref37] Zakariya Y. F. (2019). Study approaches in higher education mathematics: investigating the statistical behaviour of an instrument translated into Norwegian. Educ. Sci. 9:191. doi: 10.3390/educsci9030191

[ref38] Zakariya Y. F. (2022). Cronbach's alpha in mathematics education research: its appropriateness, overuse, and alternatives in estimating scale reliability. Front. Psychol. 13:1074430. doi: 10.3389/fpsyg.2022.1074430, PMID: 36619096 PMC9813591

[ref39] Zakariya Y. F. Massimiliano B. (2022). Short form of revised two-factor study process questionnaire: development, validation, and cross-validation in two European countries. Stud. Educ. Eval. 75:101206. doi: 10.1016/j.stueduc.2022.101206

[ref40] Zakariya Y. F. Midttun Ø. Nyberg S. O. G. Gjesteland T. (2022a). Reforming the teaching and learning of foundational mathematics courses: an investigation into the status quo of teaching, feedback delivery, and assessment in a first-year Calculus course. Mathematics 10:2164. doi: 10.3390/math10132164

[ref41] Zakariya Y. F. Nilsen H. K. Bjørkestøl K. Goodchild S. (2020). Impact of attitude on approaches to learning mathematics: A structural equation modelling approach Third conference of the international network for didactic research in university mathematics, Bizerte, Tunisia.

[ref42] Zakariya Y. F. Nilsen H. K. Bjørkestøl K. Goodchild S. (2021). Analysis of relationships between prior knowledge, approaches to learning, and mathematics performance among engineering students. Int. J. Math. Educ. Sci. Technol. 54, 1–19. doi: 10.1080/0020739x.2021.1984596

[ref43] Zakariya Y. F. Nilsen H. K. Goodchild S. Bjørkestøl K. (2022b). Self-efficacy and approaches to learning mathematics among engineering students: empirical evidence for potential causal relations. Int. J. Math. Educ. Sci. Technol. 53, 827–841. doi: 10.1080/0020739X.2020.1783006

