# Supplementary material for: Approaches to learning mathematics: preliminary evidence of a concise, valid, and reliable instrument
Source: Front Psychol. 2023 Oct 18;14:1286394. doi: 10.3389/fpsyg.2023.1286394 (PMC10619651; doi:10.3389/fpsyg.2023.1286394)
Supplement: Supplementary file 1 [file Data_Sheet_1.docx]

**Appendix A**: The polychoric correlation matrix for seven-item ALMQ

|  | ALMQ01 | ALMQ02 | ALMQ03 | ALMQ04 | ALMQ06 | ALMQ07 | ALMQ08 |
| --- | --- | --- | --- | --- | --- | --- | --- |
| ALMQ01 | 1.000 |  |  |  |  |  |  |
| ALMQ02 | .542 | 1.000 |  |  |  |  |  |
| ALMQ03 | .479 | .509 | 1.000 |  |  |  |  |
| ALMQ04 | .489 | .574 | .533 | 1.000 |  |  |  |
| ALMQ06 | -.180 | -.149 | -.220 | -.181 | 1.000 |  |  |
| ALMQ07 | -.150 | -.173 | -.197 | -.147 | .542 | 1.000 |  |
| ALMQ08 | -.198 | -.258 | -.223 | -.201 | .491 | .558 | 1.000 |

**Appendix B: Approaches to Learning Mathematics Questionnaire**

This questionnaire contains some items about your studies and the way you normally study mathematics. There is no right way to study. It depends on what suits you and what subjects you study. Therefore, it is important that you answer each question as honestly as possible.

Please, fill in the appropriate tick in the box provided alongside the item statement. The letters alongside each item statement stand for the following response.

A—This item is *never* or *only rarely* true of me

B—This item is *sometimes* true of me

C—This item is true of me about *half the time*

D—This item is *frequently* true of me

E—This item is *always* or *almost always* true of me

| Item | Item statement | A | B | C | D | E |
| --- | --- | --- | --- | --- | --- | --- |
| 1 | I feel that virtually any topic in mathematics can be highly interesting once I get into it. |  |  |  |  |  |
| 2 | I find most new topics in mathematics interesting and often spend extra time trying to obtain more information about them |  |  |  |  |  |
| 3 | I test myself on important topics in mathematics until I understand them completely. |  |  |  |  |  |
| 4 | I work hard to study mathematics because I find the material interesting. |  |  |  |  |  |
| 5 | I find it is not helpful to study mathematics topics in depth. It confuses and wastes time when all you need is a passing acquaintance with topics in mathematics. |  |  |  |  |  |
| 6 | I believe that teachers should not expect students to spend significant amounts of time studying mathematics material everyone knows will not be examined. |  |  |  |  |  |
| 7 | I see no point in learning mathematics material which is not likely to be in the examination. |  |  |  |  |  |

1. **Gender**: Male Female
2. **Course of Study** …………………………………………………
3. **Age**: ……………. Years

**Deep approaches to learning mathematics**: Item 1 – Item 4

Surface approaches to learning mathematics: Item 5 – Item 7.
